# Using the Revised Diagnostic Criteria for Frontotemporal Dementia in India: Evidence of an Advanced and Florid Disease

**DOI:** 10.1371/journal.pone.0060999

**Published:** 2013-04-15

**Authors:** Amitabha Ghosh, Aparna Dutt, Madhura Ghosh, Pallavi Bhargava, Sulakshana Rao

**Affiliations:** Department of Neurology and Cognitive Neurology Unit, Apollo Gleneagles Hospitals, Kolkata, India; University of Manchester, United Kingdom

## Abstract

**Background:**

The International Consortium (FTDC) that revised the diagnostic criteria for behavioural variant frontotemporal dementia (bvFTD) did not have an Asian representation. Whether the revised criteria are equally useful in the early detection of Asian bvFTD patients therefore remains largely unexplored. Earlier studies have indicated differences in clinical manifestations in Indian and other Asian bvFTD patients when compared to western groups. There is an urgent need for clarification, given the projected exponential rise in dementia in these countries and the imminent clinical trials on bvFTD.

**Objective:**

To assess how Indian bvFTD patients fulfil the FTDC criteria, hypothesizing that our patients might present differently early in the illness.

**Method:**

In a hospital-based retrospective observational study, we assessed 48 probable bvFTD patients, diagnosed according to the FTDC criteria, for the speed with which these criteria were fulfilled, the frequency of individual symptoms and their order of appearance during the illness.

**Results:**

Most of our patients presented with moderate to severe dementia, in spite of having relatively short onset to diagnosis times. Patients on average took 1.4 years from onset to meet the FTDC criteria, with 90% of them presenting with four or more symptoms at diagnosis. Disinhibition was the commonest symptom and the first symptom in most patients.

**Conclusion:**

With most patients presenting with advanced and florid disease, the FTDC criteria have little additional impact in early identification of bvFTD in India. Modifying the criteria further could allow detection of Indian patients early enough for their inclusion in future clinical trials.

## Introduction

The behavioural variant of frontotemporal dementias (bvFTD) is characterized by personality and behavioural changes with relative preservation of episodic memory and visuospatial function. Since 1998, the Consensus criteria have been the cornerstone of diagnosis for bvFTD. [1] The criteria require fulfilment of all of five core diagnostic features at presentation and have a diagnostic specificity of 90–100%. [2], [3] However, this rigid requirement, together with a sizeable exclusion criteria, lowers the sensitivity of the 1998 criteria to between 33% and 79% in the early part of the illness. [2], [4] To address this issue, an international bvFTD criteria Consortium (FTDC) proposed new and highly sensitive revised criteria for the diagnosis of bvFTD. [5] The consortium divided bvFTD into three clinical groups, possible, probable and definite bvFTD, determined by a combination of clinical symptoms, imaging and pathology or genetic data. However, this globally impactful study did not have an Asian representation. Whether the revised criteria are equally useful in the early detection of Asian bvFTD patients therefore remains largely unexplored. Cross-cultural differences have been described in bvFTD, with some Asian studies showing an advanced disease at presentation in their patients.[6]–[9] If confirmed, these differences could impact future clinical trials of bvFTD. Of immediate relevance is the projected large and exponential rise in dementia patients in India and other Asian countries. [10].

We therefore proceeded to study the patterns in which Indian patients fulfil the FTDC criteria for bvFTD, hypothesizing that our patients might have a more advanced and florid disease, early in the illness.

## Methods

### Ethics Statement

The study was approved by the Local Ethics Committee, Apollo Gleneagles Hospitals, Kolkata. All patients or their authorized signatories had given written informed consent for research at the time of first presentation.

### Patient Selection

Case records of all patients referred by local neurologists or psychiatrists to the cognitive neurology clinic of our hospital between 2007 and 2012 with an initial suspicion of bvFTD were reviewed. An expert cognitive neurologist and an expert neuropsychologist independently scrutinized the records for unequivocally recorded evidence of disease progression and any of the six clinically discriminating features of the FTDC criteria for ‘possible’ bvFTD. [5] The scrutinized documents included the history and clinical examination sheets, records of caregivers’ observations, behavioural questionnaires and neuropsychological test records. Only those symptoms that had already developed by the time of presentation were considered. For each type of behaviour that was present, the examiners tried to determine its exact nature and its time of occurrence relative to the onset of the illness. Insufficient or doubtful data were strictly rated as ‘absent’. Inter-rater reliability for the diagnosis of possible bvFTD was high (κ = 0.87). Disagreements between the raters were resolved through consensus. Patients who had three or more cardinal FTDC features were then checked for evidence of functional decline and imaging evidence of frontal or temporal atrophy (MR or CT) or hypoperfusion (SPECT) to identify those with ‘probable’ bvFTD. A cognitive neurologist (AG) with extensive experience in reading imaging in dementia patients reviewed the scan records. Many of our patients had already had their imaging done before being referred to us. These were both diverse and frequently done in centres with non-standardized protocols. Therefore, only gross and visually unequivocal regional involvements on either side, for example, in the frontal, insular, anterior temporal, medial temporal, parietal or subcortical regions were considered, to maintain uniformity.

Patients were excluded if they had any psychiatric disorder, or clinical or imaging evidence of another disorder that could explain their symptoms, or had no imaging done at all, or if they had any of the exclusion features of the 1998 criteria. Out of 67 patients who had a history of progression and three or more of the clinically discriminating symptoms of the FTDC criteria, 17 patients were excluded for the following reasons: uncertain dates of onset of individual symptoms (6 patients), no imaging records available (5 patients), imaging evidence of infarcts in the frontal lobes or frontostriatal pathways (4 patients), frontal meningioma (one patient) and history of recurrent hypoglycaemic episodes (one patient). The remaining 50 patients fulfilled the FTDC criteria for possible bvFTD. Forty-eight of these patients (31 men; 17 women) also met the criteria for probable bvFTD and were taken up for further analyses. Observations included the speed with which the FTDC criteria were fulfilled, the frequency of individual symptoms and their order of appearance during the illness. The presence of core and supportive features of the 1998 criteria was also explored in them at this stage. As many of these patients were lost to follow up, an attempt was made to telephonically contact their caregivers for any brief clinical update and to check for non-survivors.

The study was approved by the Local Ethics Committee, Apollo Gleneagles Hospitals, Kolkata. All patients or their authorized signatories had given written informed consent for research at the time of first presentation.

### Statistics

Frequencies are used to describe categorical variables and mean (SD) are used for continuous variables. Mann-Whitney U test was used to compare continuous variables in survivors and non-survivors. Statistical analyses were done using the SPSS 16 for Windows (SPSS Inc., Chicago, IL) and Graphpad Instat software version 3.10 (Graphpad Software Inc. San Diego, CA). Statistical analyses was 2-tailed and *p*<0.05 was considered statistically significant.

## Results

Age of onset of illness varied between 44 years and 74 years. Mean (SD) age of onset of these patients was 60 (7.4) years and the mean onset-to-diagnosis time was 2.9 (1.6) years. Onset at or above 65 years of age was seen in 29% of our patients. Mean Bengali Mini Mental State Examination score (BMSE) [11] was 17.1 (7.7) for 40 patients in whom the scores were available. Global clinical dementia rating (CDR) [12] scores were as follows: CDR  = 0.5 in 4%; CDR = 1 in 38%; CDR  = 2 in 33%; CDR = 3 in 25%. Patients received a mean (SD) formal education of 11.9 (4.8) years. Family history of any frontotemporal lobar degeneration disorder was present in 16.7% of patients, with first-degree relatives affected in 8.3% of patients. Only one patient had similar illness in two first-degree relatives. Imaging evidence of predominant frontal or frontotemporal atrophy was reported mainly in the right hemisphere in 23% of patients, in the left hemisphere in 8% patients and bilaterally in 69% of patients. Twelve patients, out of the 35 whose families could be contacted, had died. The mean duration of the illness for these patients was 5.4 (2.6) years from onset to death and 2.8 (1.8) years from diagnosis to death. BMSE scores and global CDR scores at presentation, estimated age of onset and onset-to-diagnosis times in the patients who died and in those who continue to survive were not significantly different.

**Figure 1:**
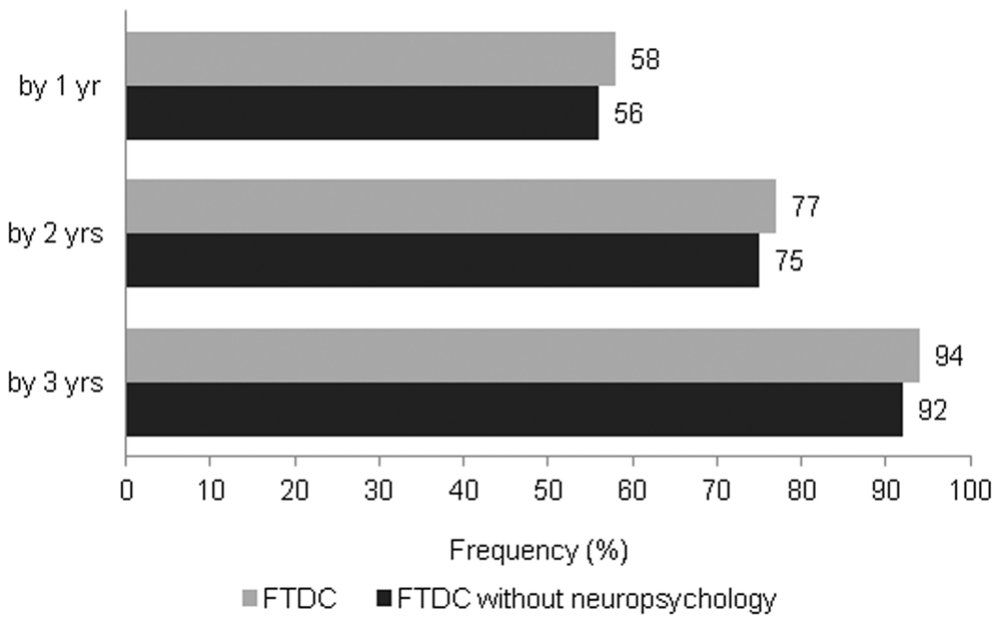
Percentage patients meeting diagnostic criteria for bvFTD at different times from onset of illness. The number at the end of the bars represent the percentage of patients who meet the FTDC criteria for bvFTD, firstly, with the option to use neuropsychological tests, if needed (grey bars), and then, without this option being provided (black bars). Percentage of patients meeting the criteria at 1 year, 2 years and 3 years from onset of illness are recorded.

By the time they presented, 43 among the 48 patients (90%) had already met at least four of the six clinically discriminatory features of the FTDC criteria whereas 28 patients (58%) met at least five of these features. All of the six features were present in 17% of patients. Patients on an average required 1.4 (1.2) years from onset to meet the FTDC criteria. Twenty-eight patients fulfilled at least three clinically discriminating features of the FTDC criteria by one year of onset, 37 patients by two years and 45 patients by three years of onset. Notably, 45 out of 48 patients (94%) fulfilled the FTDC criteria even when the neuropsychological criterion was excluded from consideration. (Figure 1).

**Figure 2:**
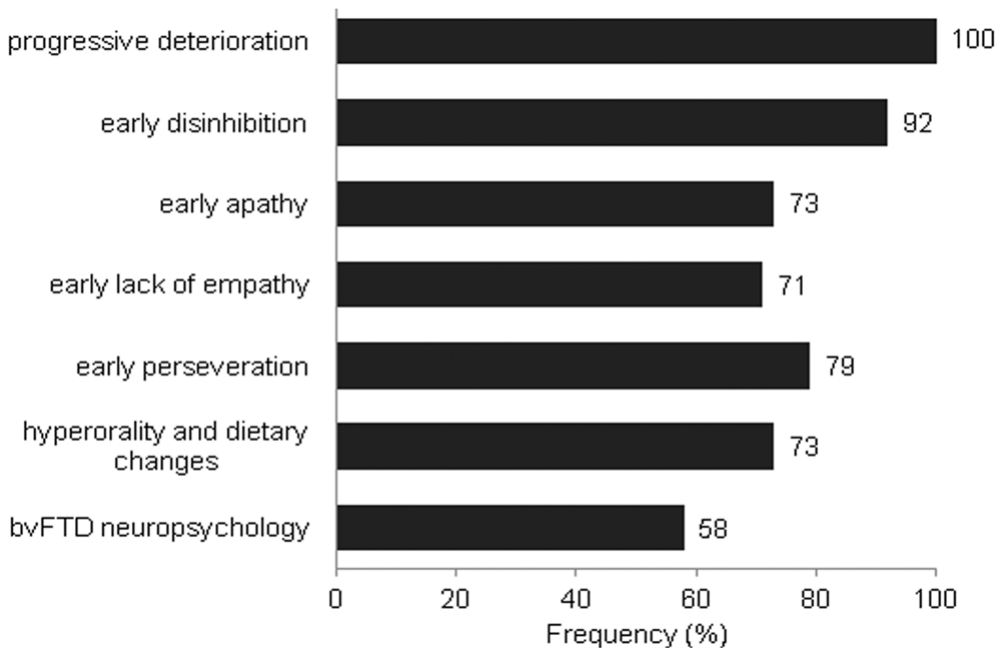
Frequency of cardinal features of the FTDC criteria. The numbers at the end of the bars represent percentage frequencies. Neuropsychology assessment was consistent with the diagnosis of bvFTD in all 58% patients where it could be done.

**Figure 3:**
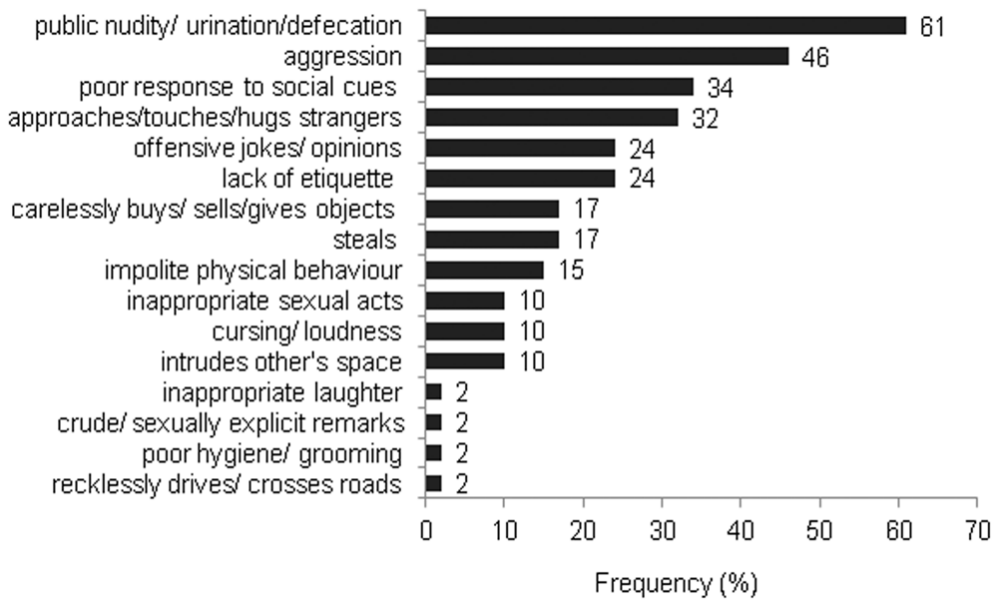
Frequency of occurrence of different subtypes of disinhibition. (No separate legend for this figure).

Figure 2 shows the frequency of appearance of the FTDC symptoms in our patients. Early disinhibition (44 patients) was most frequent, with public nudity, or urinating or defecating in public places, being the commonest type, followed by physical or verbal aggression.(Figure 3) Patients exhibited 16 different types of disinhibition with an average of 2.5 (1.2) of them occurring within one year of illness. Fifty-eight percent of patients had undergone neuropsychological tests for executive functions, episodic memory and visuospatial functions at the time of first presentation. All of them showed predominant executive dysfunction, evidenced by impairment in at least two among the tests of verbal fluency (semantic and phonemic), backward digit span and Wisconsin Card Sorting Test [13] that have been adapted and validated for Indian patients. [11], [14] Episodic memory and visuospatial functions were largely preserved.

**Figure 4:**
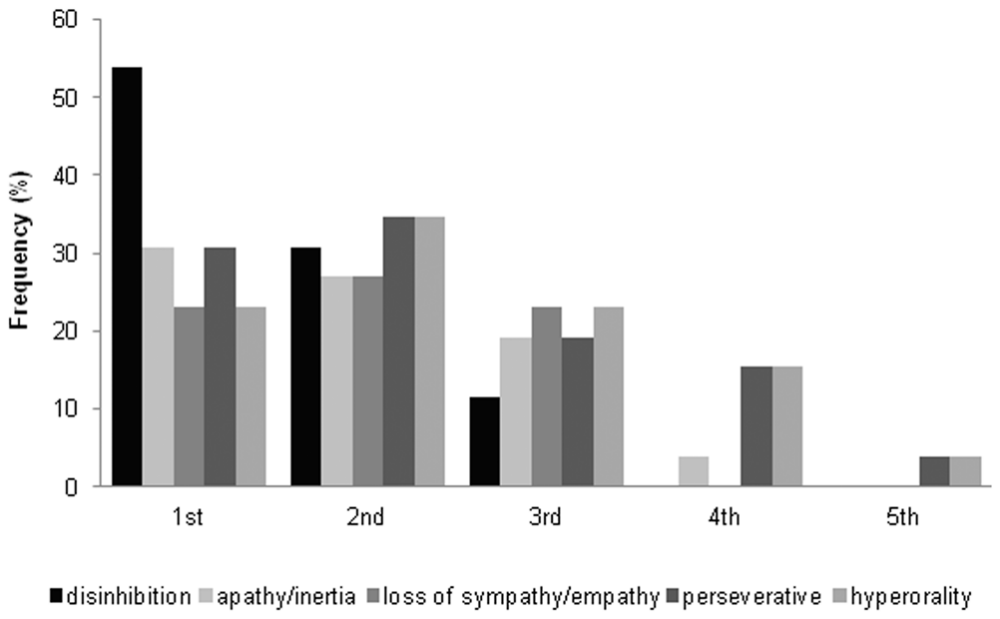
Order of appearance of symptoms of the FTDC criteria and their frequency. The horizontal axis denotes the order of appearance of symptoms.

Figure 4 shows the order of appearance of the cardinal symptoms of the FTDC criteria in the 29 patients where unambiguous sequential information was available. Disinhibition was the first symptom in 54% patients and was always among the first three symptoms of the disease. Loss of sympathy or empathy was also among the first three symptoms but unlike disinhibition, was more evenly distributed in this space. Apathy was usually among the first three symptoms but occasionally presented as the fourth symptom. Perseverative behaviour and hyperorality presented throughout the illness although mostly occurring among the first four symptoms.

Forty-five patients (94%) with probable bvFTD met all of the core features of the1998 criteria at presentation, taking an average of 1.8 (1.6) years from onset to do so. Forty-seven percent of those who met the 1998 criteria did so within one year of illness. In addition, the patients also had an average of seven supportive features. To avoid circularity caused by patient selection, we excluded the following supportive features from further analysis: hyperorality and dietary changes, perseverative and stereotyped behaviour, stereotypy of speech, perseverative speech, neuropsychological findings and imaging findings of typical frontal or anterior temporal involvement. In spite of this truncation, patients continued to demonstrate an average of three supportive features of the 1998 criteria. Utilization behaviour was the most common (77%), followed by distractibility (48%) and mental rigidity (40%). Echolalia (41%) was the commonest speech disorder seen in our patients. Physical signs were less frequent, with incontinence reported in 27% of patients.

## Discussion

By the time they presented, 90% of our patients had already fulfilled four or more of the FTDC criteria, thereby overshooting its minimum requirements. In comparison, the large FTDC study itself, done mainly on western European and North American bvFTD patients, identified only 69% patients who exhibited similar findings [5]. Our results could have two potential implications. Either our patients presented late into their illness, or, they could have a more florid clinical presentation, even early in the course. The mean onset-to-diagnosis time of less than three years in our patients is lower than what has frequently been reported in the western literature,[2], [5], [15]–[22] thereby, ruling out a late presentation. Many of our patients had already fulfilled the FTDC criteria long before they were formally diagnosed. Almost 60% of them did so within one year of symptom onset. Additionally, a little less than half of the patients fulfilled the 1998 criteria by the first year and nearly 90% of them did so by three years of onset. This is contrary to reports of poor sensitivity of the 1998 criteria that has largely emerged from the western literature [4], [5], [23] and suggests a more developed early clinical pattern in our patients.

A striking feature of our study was the fulfilment of the FTDC criteria in most patients by symptoms alone, even without having to test for executive functions. Besides highlighting the floridity of symptoms, this also benefits patients who cannot undergo neuropsychological tests, either due to lack of adequately trained raters in many parts of our country, or due to the severity of their dementia.

Indeed, most of our patients already had moderate to severe dementia at the time of diagnosis. In comparison, patients from western Europe or North America appear to present with much milder disease.[5], [15], [17]–[21], [24], [25] Interestingly, demographic details of patients in other Asian studies on bvFTD have frequently shown them to have a more advanced disease at presentation, even though this was not always highlighted by the authors.[7], [26]–[32] For example, in a study comparing eating behavior in bvFTD patients from Japan and the UK, the average Mini-Mental State Examination (MMSE) [33] score in Japanese patients was 16.1, versus 22.9 in the UK group. [32] In another study, 37 Japanese bvFTD patients had MMSE scores of 18.7 (5.3) at presentation. [30] A recent systematic review from Hong Kong identified 35 bvFTD patients of Han Chinese ethnicity. Their mean MMSE score was 13.9 (6.7). [7] The consistently low scores in our patients in spite of shorter onset-to-diagnosis times parallel results from other Asian studies, as opposed to those reported in western European and North American literature.[5], [8], [15], [17]–[19], [21], [24]–[26], [29], [34], [35] A recent cross-cultural study comparing US patients with Greek and Turkish patients also reported similar findings. [6] There are several possible explanations for this pattern. Families of Asian patients, like those in Greece and Turkey, could be mistaking early symptoms for routine consequences of normal ageing. [6], [31] However, it is unlikely that experienced cognitive neurologists in these countries would consistently fail to estimate a reasonable time of symptom onset. For example, florid disinhibition would be difficult to pass as normal behaviour for long. Early disinhibition was almost universal in our patients and was more common than apathy. It was also the commonest first symptom volunteered by the family who, on average, reported two to three varieties of disinhibition developing within the first year of illness alone. When assessing behavioral change with respect to the premorbid state, behaviours like public nudity, urination or defecation in public places, physical or verbal aggression, or touching or hugging strangers, would be difficult to miss. Further evidence of a more rapidly progressive disease comes from the mortality reports in our patients. The relatively short disease duration until death in spite of comparable dementia severity at diagnosis, age at onset and onset-to-diagnosis time between the survivors and non-survivors indicates a clinical decline in our patients that is faster than that described in the literature. [5], [19].

Family history was uncommon in our patients, unlike in the ‘western’ groups, where a family history of FTD spectrum disorder is reported in about 30–50% of patients.[36]–[38] Our results are closer to other Asian reports, most of which describe family history in less than 30% of bvFTD patients. Japanese researchers, for example, failed to elicit a strong family history from patients with bvFTD. [27], [39] A Chinese study found that only 20% of bvFTD patients had a positive family history. [7] A preliminary study of the genetics of FTD in Indian patients did not find any pathogenic mutation in the genes encoding for microtubular associated protein tau (*MAPT*) or progranulin (*PGRN*). [40] More extensive genetic studies are in progress.

Put together, the demographic features that distinguished our bvFTD patients from the western European and North American groups, while reflecting a possible common pattern in Asian patients include 1) moderate to severe dementia at the time of diagnosis, 2) relatively short onset-to-diagnosis time and 3) relatively weak family history. Although prominent and early disinhibition occurred in most of our patients, we acknowledge that other Asian groups may have a predominantly apathetic variant instead. [28] Our patients exhibited florid clinical symptoms in spite of early fulfilment of clinical criteria. Except for one study with similar findings, Asian data in this regard is limited. [26] Indeed, many of our patients fulfilled the FTDC as well as the 1998 criteria well before they presented to our clinic. Furthermore, a rapid progression to death was seen in our patients. Among other known distinguishing symptoms, our bvFTD patients have consistently exhibited a high frequency of utilization behaviour and imitation behaviour. [8], [9] A Japanese study also demonstrated frequent imitation behaviour in bvFTD patients. [30] Although their relative rarity in western European and North American bvFTD patients could reflect a less focused search for these behaviours [9], the existence of a distinct behavioural pattern in Asian patients cannot be ruled out. Clearly, many more studies looking at clinical patterns, genetics and socio-cultural influences from across the Asian region are needed for more definitive patterns to be recognized.

The absence of pathological diagnosis limited our study. However, the fact that most of our patients who fulfilled the FTDC criteria for probable bvFTD also fulfilled the 1998 consensus criteria suggests a high level of diagnostic specificity. Indeed, the purpose of our study was neither to identify new diagnostic criteria, nor to assess the sensitivity or specificity of the existing ones, but to see how the best-recognized core clinical features of bvFTD are represented in Indian patients. Our study was retrospective and some of the clinical data might have been under-represented. For example, when a symptom was in doubt or not recorded, it was marked as absent. However, considering that assessments done in our cognitive clinic follow a set routine, these are likely to be true representations of the clinical findings. Similarly, the variable types and quality of imaging available to us precluded a useful clinico-radiological interpretation. We are working on future prospective studies to address these issues.

Nearly 4 million Indians suffer from dementia today and the numbers are expected to double in the next 20 years. [10] With increasing interest in clinical trials for bvFTD, the identification of patients with relatively mild disease is crucial. Unfortunately, many of our patients who are diagnosed on the basis of the FTDC criteria already have a moderate to severe disease at the time of diagnosis. Therefore, selecting patients on the basis of these criteria might be too late for our patients. The possibility of modifying the FTDC criteria to include less than three clinically discriminating symptoms, while increasing the role of tests for early detection for social cognition deficits, or of more specific brain imaging or other biomarkers, for example, are areas that need urgent attention.
